# Carbon Black

**DOI:** 10.1289/ehp.1103444

**Published:** 2011-08-01

**Authors:** Robert J. McCunney, Peter Morfeld, Len Levy, Henry Muranko

**Affiliations:** Massachusetts Institute of Technology, Cambridge, MA, E-mail: mccunney@mit.edu; Institute for Occupational Medicine at Cologne University, Cologne, Germany; Emeritus Professor, Institute of Environment and Health, Cranfield University, Cranfield, Bedfordshire, United Kingdom; Muranko and Associates, Scottsdale, Arizona

In “Research Recommendations for Selected IARC-Classified Agents,” Ward et al. (2010) identified research gaps for 20 occupational agents “based on evidence of widespread human exposures and potential carcinogenicity in animals or humans.” (Ward et al. 2010) For carbon black, the authors  suggested that

Research needs include updating epidemiology cohorts with data on work histories and exposures in relation to particle size and surface area, and recruitment of additional carbon black facilities. The relationship between occupational exposure to carbon black and validated biomarkers of oxidative stress should be examined and exposure–response relationships in humans and rodents quantified, including the role of particle size.

Ward et al. (2010) referred to a study of British carbon black workers in which carbon black was suggested as a possible “late stage carcinogen” (Sorahan and Harrington 2007). In that study, Sorahan and Harrington (2007) called for similar analyses of other carbon black cohorts (i.e., evaluating the possibility of carbon black acting as a late stage carcinogen via the concept of “lugging,” which considers only recent exposures and not historical exposures). In response to suggestions made by Sorahan and Harrington, we conducted such analyses on a large German carbon black cohort (Morfeld and McCunney 2007, 2009). We were unable to reproduce the results of the British analysis, despite the elevation noted in lung cancer among German cohort workers, thus providing no support for the late stage-lugging hypothesis. Results of a detailed analysis of the German cohort using Bayesian methodology showed smoking and exposure to occupational carcinogens prior to work at the carbon black plant as confounders probably responsible for the lung cancer excess (Morfeld and McCunney 2010).

Ward et al. (2010) called for enhanced exposure–response assessments in humans. Currently, a dose–response exposure analysis is under way on the U.S. carbon black cohort (> 5,000 production workers). An earlier evaluation of this cohort showed no increase in any type of cancer (Dell et al. 2006).

Ward et al. (2010) recommended that “the relationship between occupational exposure to carbon black and validated biomarkers of oxidative stress should be examined.” Despite the appeal of biomarkers of oxidative stress in pinpointing inflammatory changes associated with malignant and nonmalignant illnesses, such markers are nonspecific, not well validated, and appear not “ready for prime time,” as noted in a recent symposium on nanotoxicology (Fischman et al. 2011).

A meta-analysis of all three major carbon black cohorts (United States, United Kingdom, and Germany) to assess risk of heart disease is also under way. In a recent position paper, Brook et al. (2004) noted that particle exposure may play a role in the development of heart disease.

Ward et al. (2010) suggested evaluating carbon black particle size and surface area. However, the physical and chemical properties of untreated manufactured carbon blacks are distinctly different from ubiquitous carbon core particulates in both occupational and ambient atmospheres (Kuhlbusch and Fissan 2006). Approximately 90% of manufactured carbon black is used for tire and automotive rubber products. In products, such as toners, plastics, and surface coatings, carbon black is matrix-bound, and not an exposure risk to end-users. Care should be taken when applying quantitative models that claim to address the particle size and surface area topics (Tomenson and Morfeld 2010).
